# Supplementary figures and images for: A Triazaspirane Derivative Inhibits Migration and Invasion in PC3 Prostate Cancer Cells
Source: Molecules. 2023 Jun 2;28(11):4524. doi: 10.3390/molecules28114524 (PMC10254162; doi:10.3390/molecules28114524)

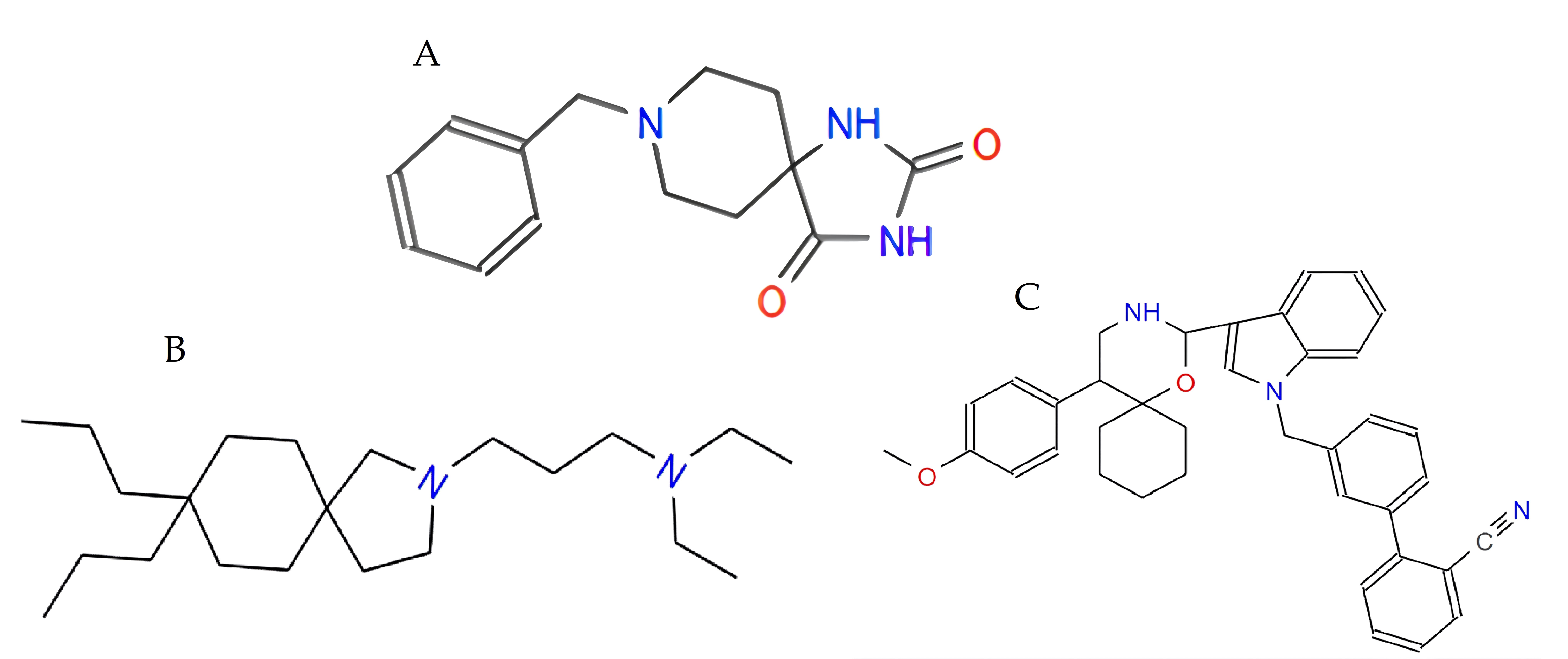

Supplement: Supplementary file 1 [file molecules-28-04524-s001.zip › molecules-2410204-supplementary.jpeg]
